# Strategies for Optimizing the Production of Proteins and Peptides with Multiple Disulfide Bonds

**DOI:** 10.3390/antibiotics9090541

**Published:** 2020-08-26

**Authors:** Yunqi Ma, Chang-Joo Lee, Jang-Su Park

**Affiliations:** Department of Chemistry and Chemistry Institute of Functional Materials, Pusan National University, Busan 609-735, Korea; michell@pusan.ac.kr (Y.M.); gd0090s@naver.com (C.-J.L.)

**Keywords:** multiple disulfides, expression optimization, leading protein, signal peptide, yeast, high yield

## Abstract

Bacteria can produce recombinant proteins quickly and cost effectively. However, their physiological properties limit their use for the production of proteins in their native form, especially polypeptides that are subjected to major post-translational modifications. Proteins that rely on disulfide bridges for their stability are difficult to produce in *Escherichia coli*. The bacterium offers the least costly, simplest, and fastest method for protein production. However, it is difficult to produce proteins with a very large size. *Saccharomyces cerevisiae* and *Pichia pastoris* are the most commonly used yeast species for protein production. At a low expense, yeasts can offer high protein yields, generate proteins with a molecular weight greater than 50 kDa, extract signal sequences, and glycosylate proteins. Both eukaryotic and prokaryotic species maintain reducing conditions in the cytoplasm. Hence, the formation of disulfide bonds is inhibited. These bonds are formed in eukaryotic cells during the export cycle, under the oxidizing conditions of the endoplasmic reticulum. Bacteria do not have an advanced subcellular space, but in the oxidizing periplasm, they exhibit both export systems and enzymatic activities directed at the formation and quality of disulfide bonds. Here, we discuss current techniques used to target eukaryotic and prokaryotic species for the generation of correctly folded proteins with disulfide bonds.

## 1. Introduction 

Proteins play a major role in cell signaling, inflammatory reactions, cell adhesion, and cell cycle. Commercially, they are primarily produced with the aid of genetic and protein engineering [1]. Native and recombinant proteins are used in a wide range of areas in the biopharmaceutical, enzyme, and agricultural industries. Such molecules are increasingly being used in the fields of pharmacy, diagnostics, milk, diet, detergents, textiles, clothing, paper, pulp, polymers, and plastics [2,3,4,5,6].

**Figure 1:**
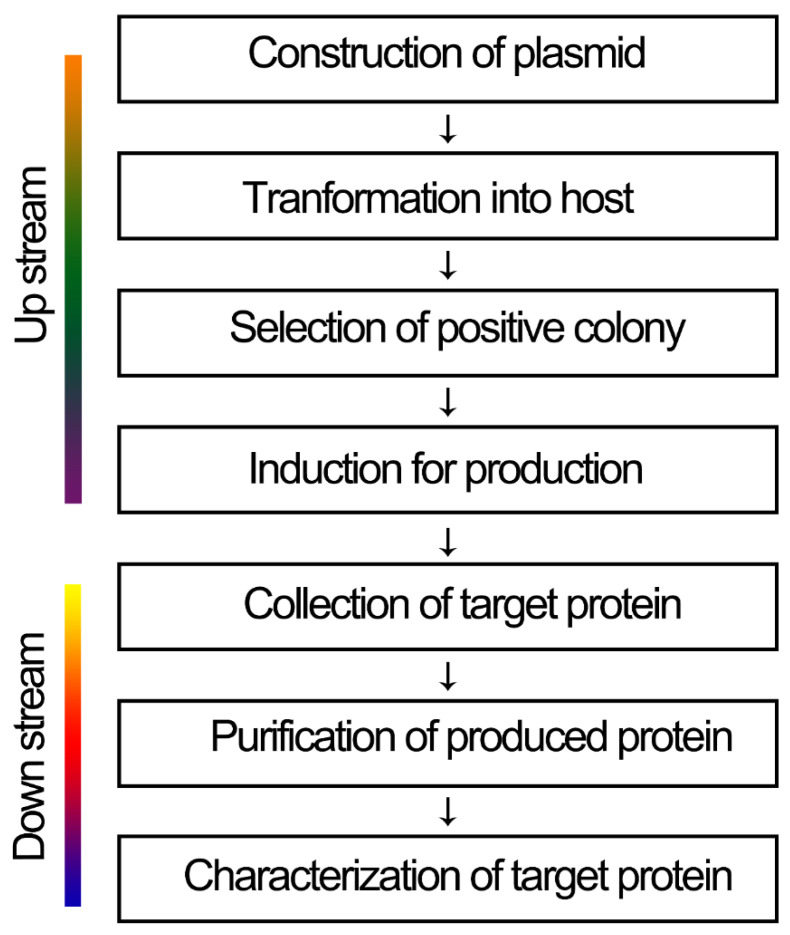
Recombinant protein production process. The process includes upstream and downstream.

In conventional biotechnology, cells have been used for several processes. Across all genetics and molecular biology revolutions to date, recombinant protein processing is a significant method that requires the use of cells, and it is a state-of-the-art method for pharmaceutical protein processing and screening [6]. The bioprocess for recombinant protein production (Figure 1) is divided into upstream and downstream sequences: The cells are fermented before and during protein production (upstream), and the product is either excreted from the cells into the culture medium or the cells are lysed and the cell lysates are processed further (downstream) [7]. Downstream processes may include the separation of soluble and insoluble cell debris and media components, protein purification, protein formulation (including concentration), bioconjugation, protein-bioconjugate purification, and protein refolding. Only one downstream process is required depending on the protein and its specific requirements [8]. Proteins are susceptible to denaturation by several factors, such as pH, salt concentration, organic solvents, shearing, surface and interface interaction (including the formation of protein aggregates), lyophilization, moisture levels, protein concentration, and temperature changes. Therefore, such processes need to be carefully engineered to generate intact and functional proteins [9,10].

Large proteins (usually more than 400 amino acids) are typically expressed in a eukaryotic system, whereas smaller ones are expressed in prokaryotic systems. Mammalian cells, fungi, or the baculovirus system are typically chosen for proteins requiring glycosylation. The least expensive, simplest, and fastest protein expression can be achieved in *Escherichia coli*. Nonetheless, large proteins are difficult to be synthesized with this bacterium. Moreover, *E. coli* is not the best system for S–S-rich proteins that require post-translational modifications. *Saccharomyces cerevisiae* and *Pichia pastoris* are the most frequently used yeast species for the production of such proteins. At a low cost, yeast systems typically generate high protein yields as well as proteins with a molecular weight greater than 50 kDa, along with the extraction of signal sequences and stimulation of glycosylation. The baculovirus system can incorporate more complex post-translational protein modifications. Most commonly, mammalian cells are used for the production of recombinant mammalian glycosylated proteins. Genetically engineered animals secrete recombinant proteins in their milk, blood, or urine. Similarly, several recombinant proteins can be produced from transgenic plants, such as *Arabidopsis thaliana*. 

**Figure 2:**
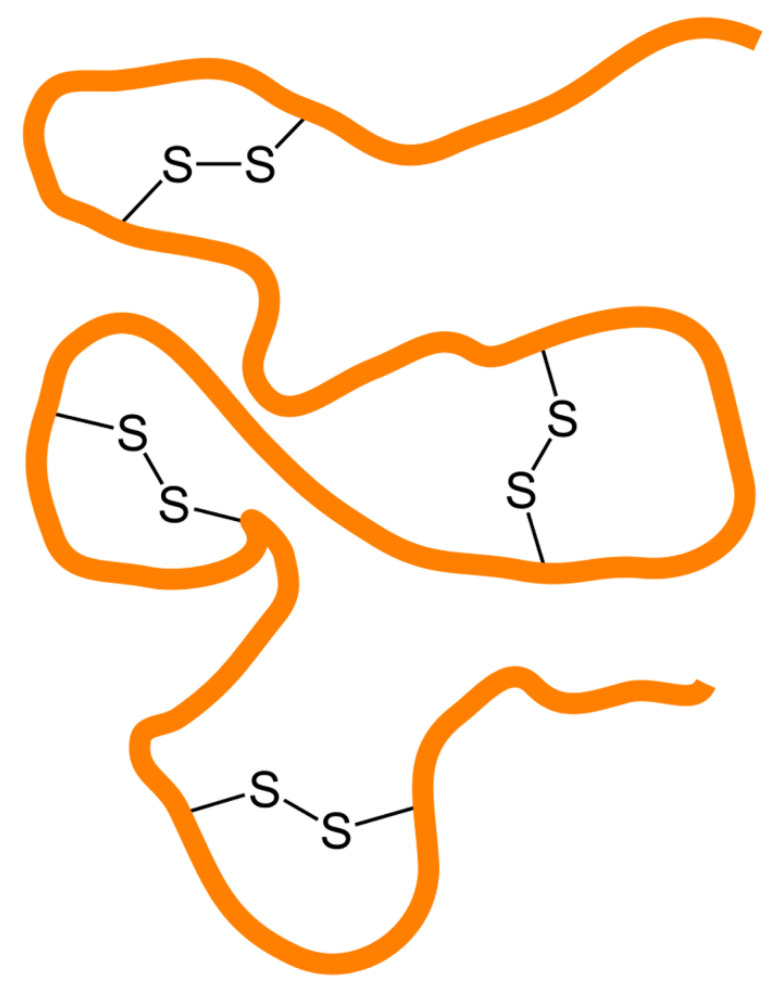
Schematic of disulfide bonds crosslinking regions of a protein.

The effectiveness of expressing a recombinant protein in *E. coli* primarily relies on the ability to prevent unfavorable interactions between newly expressed polypeptides. These interactions lead to the aggregation of intermediate folding substances instead of native protein production [6,7,8,11]. System efficiency can be improved by maintaining conditions that stabilize intermediate folding and promote mature structure formation. Various strategies can help prevent the aggregation of proteins by masking the hydrophobic patches on their external surfaces [12,13]. Such strategies involve the introduction of chaperone molecules, incorporation of detergents, or co-expression of interacting sub-units involved in large complexes. When the conditions are designed to maintain the mono-dispersion of the folding intermediates, speeding up the folding cycle becomes essential for the production of stable native structures and prevention of the accumulation of metastable configurations that are potentially vulnerable to aggregation [14]. Foldases and isomerases may improve the folding process considerably. In this review, we focus on the solutions that are available for improving the bacterial expression of proteins that rely on the formation of disulfide bonds in their native state. Such Cys-Cys bridges (Figure 2) block folding units into stable conformations by covalently linking residues, and their formation is necessary for the stable tertiary structure of a protein [15]. The equilibrium between reduced and oxidized cysteines is regulated by the redox conditions of cellular compartments. In eukaryotic cells, the endoplasmic reticulum (ER) is the oxidative environment in which disulfide bonds are preferentially formed [16]. Therefore, to complete their folding, polypeptides expressed in the reducing cytoplasm must be guided to the ER. The targeting of the subcellular compartment is facilitated by signal peptides fused to the amino terminal of the protein that are removed after the protein is imported into the organelle. Prokaryotes share key features of the reducing cytoplasm with the eukaryotic cells but do not exhibit structures that mimic the ER. Instead, they possess an oxidizing periplasm in which pro-peptides can be translocated with an extra N-terminal export peptide. Therefore, the expression of a eukaryotic protein in the bacterial periplasm is possible after replacing the ER with a bacterial signal sequence for translocation to the periplasm. Alternative approaches include encouraging the formation of disulfide bonds by targeting nascent polypeptides to the external medium or changing the redox conditions in the cytoplasm to a moderate oxidizing state [16,17]. Overexpression and direct fusion to chaperones, foldases, and stabilizing carriers have been studied to achieve an increase in the yields of functional proteins. Finally, in chaotropic solutions, protein aggregates can be dissolved first to achieve a monodispersed state and then used as a starting material for oxidative refolding processes.

## 2. Host Strains for the Overexpression of Target Proteins

The methylotrophic yeast, *P. pastoris*, is a useful system for the production of genetically engineered enzymes (through heterologous gene expression) for both research and industrial purposes [18]. It is considered non-toxigenic and non-pathogenic, and the Food and Drug Administration (FDA, White Oak, MD, USA) has approved several products prepared with help of this organism, and these products are generally accepted as safe substances (GRAS). In addition, it is an excellent host for the synthesis of recombinant proteins and offers additional benefits ranging from rapid growth to high cell density in an inexpensive and non-complex culture medium and facilitates the purification of recombinant proteins expressed heterologically [19,20,21].

*E. coli* expression systems are often used to produce exogenous protein on laboratory and industrial scales owing to the low cost, speed, and simplicity of cultivation [22]. *E. coli* strains DH5α and Rosetta (DE3) have been used for the cloning of genes and the expression of proteins. *E. coli* is one of the earliest and most commonly used hosts for heterologous protein production [23,24]. The advantages and disadvantages are presented in Table 1. The advantages include rapid growth, fast expression, ease of cultivation, and high product yields [25,26,27]. It is used to manufacture large quantities of commercialized proteins. This method is particularly outstanding for the functional expression of non-glycosylated proteins. *E. coli* genetics are better understood than that of any other microorganism [28]. Recent advances in the understanding of the processes involved in coding, translation, and protein folding in *E. coli* and the use of advanced genetic instruments render this bacterium more useful than ever for the production of diverse eukaryotic proteins [29,30,31,32]. Its genome can be updated rapidly and reliably with ease. The regulation of promoter elements is not complex, and the number of plasmid copies can be easily adjusted. This method often adjusts the metabolic carbon supply, prevents the absorption of amino acid analogs, establishes intracellular disulfide bonds [33], and demonstrates a reproducible performance with computer control. *E. coli* can produce recombinant proteins with up to 80% of its dry weight and withstand many environmental conditions [34].

Human insulin-like growth factor (hIGF-I) is a single-chain 70-residue therapeutic protein with three disulfide bonds within its structure. IGF-I affects cell proliferation, differentiation, and apoptosis in the long term. *E. coli* promotes the production of this kind of protein through its relative ease, low cost, and accelerated high-density cultivation. Therefore, it played an important role in the pharmaceutical industry in the last decades. 

However, *E. coli* has several disadvantages that need to be addressed for efficient protein production. High cell densities cause toxicity owing to the deposition of acetates [35,36,37]. This can be inhibited by monitoring oxygen levels. Proteins that are generated as inclusion bodies are often inert, insoluble, and require refolding. Owing to the limitation associated with the processing of proteins with multiple disulfide bonds, it is extremely difficult to refold such proteins [38,39]. The *E. coli* system generates unmodified proteins without glycosylation. Hence, some of the antibodies that are synthesized do not recognize mammalian proteins [40,41,42]. Interestingly, the non-glycosylated human tPA produced in *E. coli* was fully active in vitro. Given the absence of glycosylation, the drug exhibited a four-fold longer plasma half-life and a correspondingly higher clearance rate in animals [43,44]. The resulting quantity constituted 5–10% of total *E. coli* protein. To improve the process in *E. coli*, the following measures have been used: (i) Use of multiple promoters to control expression; (ii) use of specific host strains; (iii) co-expression of chaperones and/or foldase; (iv) reduction of temperature; (v) secretion of proteins in the periplasmic space or medium; (vi) reduction of protein synthesis; (vii) shift of the growth medium; (viii) introduction of a fusion partner; (ix) release of a protein fragment; and (x) denaturation and refolding of the protein in vitro [45]. Rapid fermentation with a cell density of *E. coli* has resulted in dry cell contents ranging from 20 to 175 g/L [46]. The issue of acetate development and toxicity can be solved by exponentially feeding glucose in the media and maintaining the real rate of growth below which the development of acetate is obtained. Yields as high as 5.5 g/L of α-consensus interferon have been achieved in the broth [47]. Long-term development of the chemostat (219 generations below a dilution rate of 0.05 h^−1^) produced an *E. coli* mutant with an increased growth rate and biomass yields, shorter lag period, lower acetate production, and increased stress resistance [48]. This strain produced high yields of heterologous secreted proteins [49]. Heterologous proteins developed as inclusion bodies in *E. coli* are inert, composite, and insoluble and typically exhibit non-native intra- and inter-molecular disulfide bonds and rare free cysteines [50]. Such bodies must be separated from the cell to derive the active protein along with the proteins solubilized by denaturants, and disulfide bonds must be extracted using reduction agents. Refolding is achieved by eliminating the denaturant and reducing agent, accompanied by the protein being renatured. Heterologous recombinant proteins may be formed in a highly soluble biologically active form when their genomes are fused to the *E. coli* genes with thioredoxin [51]. Few fusions maintain the properties of thioredoxin that are produced by the methods of osmotic shock or freeze/thaw, along with strong thermal stability. Thioredoxin has a low molecular size (11 kDa) and is typically derived in a soluble form at 40% of the total cell protein [52]. Another effective approach for the development of inclusion bodies that comprise heterologous proteins is to lower the growth temperature from 37 to 30 °C [53,54]. Higher yields are usually obtained in the cytoplasm than in the periplasmic space [55]. It is necessary to export cytoplasmic proteins for the ease of the purification process and promote proper folding. This can be achieved with proteins comprising disulfide bonds since the cytoplasm eliminates an atmosphere to a great extent [56]. The fusion is achieved with a leader peptide at the N-terminal to secrete certain proteins into the periplasm. Osmotic shock or cell wall permeabilization is used to draw the proteins out of the periplasm and into the medium [57]. A mechanism involving regulation with promoter elements (lac, tac, and trc) is employed to increase the output. Promoter structures need to be defined and closely controlled to achieve a low-basal speech quality and must be easily transferable to other *E. coli* strains that require a simple and inexpensive induction technique, independent of the medium ingredients [58].

The secretion of recombinant proteins by *E. coli* into the periplasm or extracellular medium has several benefits over intracellular development as inclusion bodies. It improves packaging, folding, and in vivo downstream stability and enables the manufacture of soluble active proteins at reduced processing costs [59,60,61,62]. 

Lactic acid bacteria (LAB) comprise a large community of Gram-positive microorganisms, including members of the genera *Lactococcus*, *Lactobacillus*, *Pediococcus*, *Streptococcus*, *Leuconostoc*, and *Oenococcus*, that are used for different applications. Most of these bacteria, which have been used in food production for a long time, are deemed suitable for human consumption, and thus have GRAS status [63,64]. Owing to its simple handling, sequenced genome, and the advancement of genetic techniques in recent years, *Lactococcus lactis* is the best described species of this group and is characterized as the model organism. This has rendered *L. lactis* more suitable for biotechnological uses, ranging from recombinant protein development to the expression and distribution of antigens and bioactive polypeptides to mucosal surfaces [65], and more recently, as a distribution system for DNA vaccines [66].

Yeasts, the single-celled eukaryotic fungal cells, are also used for the development of recombinant proteins that are not well developed in *E. coli* owing to folding issues or the glycosylation requirements [67,68,69]. The major advantages of yeast expression systems are listed in Table 2. The yeast strains are genetically well defined, and several post-translation modifications have been identified [70]. They are easy to handle, less costly than insect or mammalian cells, and well suited for the fermentation processes [71]. The two most utilized yeast strains are *S. cerevisiae* and the methylotrophic yeast *P. pastoris* [72]. Specific populations of yeasts have proved to be useful in the production and study of recombinant eukaryotic proteins. Some proteins produced in different host strains are listed in Table 3. For instance, on *A. niger*, glucose oxidase can be generated by *S. cerevisiae* at 9 g/L. *S. cerevisiae* provides certain benefits as a cloning host over bacteria: (i) Traditionally, they have been used in industrial fermentation; (ii) when sufficient signal sequences have been attached to the structural genes, they can secrete heterologous proteins into the extracellular broth; and (iii) they perform protein glycosylation [73,74,75,76,77]. However, glycosylation performed by *S. cerevisiae* is sometimes unfavorable for the production of mammalian proteins as O-linked oligosaccharides often include mannose, whereas higher eukaryotic proteins produce sialylated O-linked chains [78,79]. In addition, the yeast over-glycosylates N-linked sites, which leads to reduced activity and receptor binding and may trigger an immune reaction [80]. Marketed items that are manufactured with *S. cerevisiae* include insulin, hepatitis B surface antigen, urate oxidase, glucagons, granulocyte-macrophage colony-stimulating factor (GM-CSF), hirudin, and platelet-derived growth factor [81,82].

Almost all eukaryotic polypeptides that are secreted are glycosylated. Glycosylation is species-, tissue- and cell-type specific [88,89]. In certain conditions, a protein that is typically glycosylated is activated in the absence of the carbohydrate moiety, which may be rendered in bacteria [90]. For proteins in which glycosylation is necessary for stabilization or proper folding (e.g., erythropoietin and human chorionic gonadotropin), recombinant yeast, mold, insect, or mammalian cells can be used. Mammalian cell-secreted proteins are glycosylated with D-mannose sugars covalently bound to asparagine-linked N-acetyl-D-glucosamine molecules [91,92]. Secreted fungal enzymes also exhibit the same glycosylation form [93], although additional carbohydrates connected to serine or threonine oxygen are often present in fungal proteins [94,95,96,97].

The glycosylation of a protein can differ from others based on factors, such as the medium used for culturing the cells. Glycosylation determines the kinetics of the reaction (if the protein is an enzyme), solubility, half-life serum, thermal stability, in vivo functions, immunogenicity, and binding receptors. Galactosylated enkephalins are 1000–10,000 times more soluble in peptides than the peptide alone [98,99]. The cloning of genes that encode bacterial non-glycosylated proteins in yeast results in the enhanced stability of proteins by glycosylation. The proteins produced in the yeast are glycosylated and more stable [100,101]. Glycosylation also influences the pharmacokinetics of the protein (residence time in vivo) [102,103,104]. Methods of improving the stability protect against the proteolytic assault on erythropoietin (EPO) by terminal sialic acid [105], tissue plasminogen activator (TPA) [106], and interferons. Human EPO is 1000 times more active in vivo than its desialylated form; however, both forms demonstrate identical in vitro activities [107]. Cloned glycosylated transferases will be used in the future to maintain homogeneity (“glycosylation engineering”). As hosts for the industrial development of recombinant proteins, methylotrophic yeasts have become considerably desirable, as promoters regulating the expression of genes are among the best owing to their tight control of such genes. The cells themselves can be rapidly cloned to large densities, and the degree of product expression can be controlled by basic medium manipulation. Methylotrophic yeasts can grow to a maximum density of 130 g/L [108,109]. The four recognized methylotrophic yeast genera (*Hansenula*, *Pichia*, *Candida*, and *Torulopsis*) share a specific metabolic mechanism that enables them to use methanol as their primary source of energy. Several enzymes are rapidly synthesized at high rates in a transcriptionally controlled reaction for methanol production. The ability to form disulfide bonds and protein glycosylation is the biggest advantage of *Pichia* over *E. coli*. In cases where disulfides are required, *E. coli* generates an inactive or insoluble protein that is misfolded. Compared with other expression systems, such as *Drosophila melanogaster* S2-cells or Chinese hamster ovary (CHO) cells, *Pichia* normally results in much higher yields. Cell lines derived from multicellular organisms typically need complex (rich) media, thereby increasing the cost of protein content processing. In addition, since *Pichia* can be produced in media containing only one source of carbon and one source of nitrogen, it is ideal for isotopic labeling applications, such as for performing protein NMR. Methylotrophic *P. pastoris* and its excellent capacity of protein secretion is a significant benefit for the production of recombinant proteins, as opposed to other yeasts. Progress has been achieved by genetically engineering the secretory system of *P. pastoris* for the production of human N-glycosylated proteins. Glycosylation is less extensive in *P. pastoris* than in *S. cerevisiae* due to the shorter chain lengths of N-linked high-mannose oligosaccharides, usually up to 20 residues compared to 50–150 residues in *S. cerevisiae*. *P. pastoris* also lacks α-1, 3-linked mannosyl transferase that produces α-1, 3-linked mannosyl terminal linkages in *S. cerevisiae* and leads to a highly antigenic response in patients. Hirudin, a thrombin inhibitor derived from the medicinal leech *Hirudo medicinalis*, is now produced by the recombinant yeast, *P. pastoris*, which produces high levels of mammalian recombinant proteins in the extracellular medium. An insulin precursor was produced at 1.5 g/L. Other studies demonstrate the production of 4 g/L of intracellular interleukin 2 at 30% of the total protein, 4 g/L of secreted human serum albumin, 6 g/L of tumor necrosis factor and other heterologous proteins, and 10 g/L of tumor necrosis factor. The production of serum albumin in *S. cerevisiae* amounted to 0.15 g/L, whereas the titer was 10 g/L in *P. pastoris*. *P. pastoris* produced gelatin at over 14 g/L. It also yielded 300 mg/L/day of recombinant human chitinase. Intracellular tetanus toxin fragment C was produced as 27% of the total protein with a titer of 12 g/L. Claims have been made that *P. pastoris* can produce 20–30 g/L of recombinant proteins. However, certain disadvantages have been associated with the use of *Pichia* as a host for heterologous expression. Many proteins require chaperonins for proper folding, which *Pichia* is unable to produce. Gerngross and colleagues managed to create a strain that produces EPO in its normal glycosylated form derived from humans. This was achieved by exchanging the enzymes responsible for the yeast form of glycosylation with the mammalian homologs. Thus, the altered glycosylation pattern allowed the protein to be fully functional in humans. Since then, glycosylation of recombinant proteins resulting in the human subtype that is produced in the engineered *P. pastoris* has also been demonstrated with other human proteins. Heterologous gene expression in another methylotroph, *Hansenula polymorpha*, yielded 1 g/L of intracellular hepatitis B S-antigen (50 gene copies/cell), 1.4 g/L of secreted glucoamylase (4 copies/cell), and 13.5 g/L of phytase. Secreted mammalian proteins can be produced at 3 g/L by *L. lactis*.

## 3. Location of Expression 

In principle, recombinantly expressed proteins can be directed to three different locations, namely, the cytoplasm, periplasm, or cultivation medium. Various advantages and disadvantages are associated with the direction of a recombinant protein to a specific cellular compartment (Table 4). Typically, expression in the cytoplasm is preferable as the resultant production yields are high. Disulfide bond formation is segregated in *E. coli* and is actively catalyzed in the periplasm by the Dsb system. The reduction of cysteines in the cytoplasm is achieved by thioredoxin and glutaredoxin. Thioredoxin is maintained in the reduced state by thioredoxin reductase (trxB), while glutaredoxin is maintained by glutathione. The low-molecular-weight glutathione molecule is reduced by glutathione reductase. The disruption of the *trxB* and *gor* genes that encode the two reductases allows the formation of disulfide bonds in the *E. coli* cytoplasm. The trxB and trxB/gor-negative strains of *E. coli* have been selected for the expression of several proteins. Folding and disulfide bond formation in the target protein is enhanced by fusion to thioredoxin in the strains that lack trxB. The overexpression of the periplasmic foldase, DsbC, in the cytoplasm further stimulates disulfide bond formation. Transmembrane transport is typically mediated by N-terminal signal peptides by directing the protein to a specific transporter complex in the membrane. Most proteins are exported across the inner membrane into the periplasm by the well-known Sec translocase apparatus. Frequently used periplasmic leader sequences are derived from ompT, ompA, pelB, phoA, malE, lamB, and lactamase for potential export. Systems are available for the potential export and enhanced disulfide bond formation achieved by fusion to DsbA or DsbC, which are the enzymes catalyzing disulfide bond formation and isomerization in the periplasm. A direct consequence of periplasmic production is a considerable reduction in the amount of contaminating proteins in the starting material used for purification [110]. Other benefits include a much higher probability for obtaining an authentic N-terminal in the target protein, decreased proteolysis, and simplified protein release achieved by osmotic shock procedures. Efficient pathways for translocation via the outer membrane are not known, although certain proteins that are exported to the periplasm diffuse or leak into the extracellular medium. Passive transport across the outer membrane can be stimulated by external or internal destabilization of the structural components of *E. coli* [111,112,113]. Destabilization is achieved either by the lysis of proteins functioning at the interior of the cell using strains lacking structural membrane components or permeabilization directed from the cell exterior either mechanically, enzymatic, or chemically. Another strategy involves engineering secretion mechanisms in *E. coli* by using either pathogenic *E. coli* or other species. The direction of recombinant proteins into the periplasm often results in the leakage of protein into the extracellular cultivation medium. This uncontrolled strategy enables the purification of potato carboxypeptidase inhibitor and cholera toxin B subunit. The ompA signal sequence has been recently used to translocate a recombinant peptide to the periplasm for secretion into the cultivation medium. Translocation was enhanced by the co-expression of two secretion factors along with an increase in the level of the recombinant peptide in the cultivation medium [114,115]. Recombinant proteins probably exit the periplasm passively via destabilized membrane structures, either upon cellular aging or a change in the culture conditions. However, detailed information and standardization methods for directed secretion remain unknown. 

## 4. Vector Selection for Expression 

Vector pcDNA3(−) (purchased from sigmaaldrich, Seoul, Korea South) and fusion gene were injected into the pET sequence to generate vectors for the target protein. Many genomes that were used in this analysis were designed using a similar technique. All vectors were based on the pET vector series. The propagation and amplification of the vectors were accomplished using normal techniques [116,117]. To maximize the usage of restriction locations, we decided to create two different plasmids for heavy- and light-chain expression (some vectors we used are listed in Table 5), as many sites that are rare in VH are common in VL and vice versa [118]. However, by cloning the light-chain transcription unit into the VH express backbone using *Hin*dIII and *Kpn*I or *Eco*RI, a single plasmid was formed that comprised all Ig transcription units (in either orientation with respect to each other).

## 5. Signal Peptide in Fusion Expression 

A well-designed vector for the prokaryotic expression involves a collection of optimally arranged genetic elements that influence both transcriptional and translational aspects of protein development. Additionally, the presence of an antibiotic-resistance gene promotes phenotypic vector collection, and the center of replication (Ori) defines the number of vector copies [119]. The secretion of proteins via the extracellular matrix might be a suitable technique. Similar to that in *E. coli*, this compartment exhibits the lowest degree of proteolytic activity and the purification of the potentially soluble structurally accurate protein can be significantly improved due to the presence of a few contaminating bacterial proteins. Unfortunately, this technique is not feasible for the large-scale processing of heterologous proteins in *E. coli* [120]. Proteins that are supposed to be secreted into the extracellular medium may traverse two separate membrane boundaries, namely, the cytoplasmic and the outer membranes [121]. Our knowledge of the molecular mechanisms that regulate membrane translocation, especially via the outer membrane, remains incomplete. Strategies for protein secretion in the culture medium usually fall into two categories: The use of known pathways for secreted proteins and the use of signal sequences, fusion partners, and permeabilizing agents that affect protein secretion owing to selective and restricted external membrane permeability. Protein yields corresponding to both approaches are typically low. 

**Figure 3:**
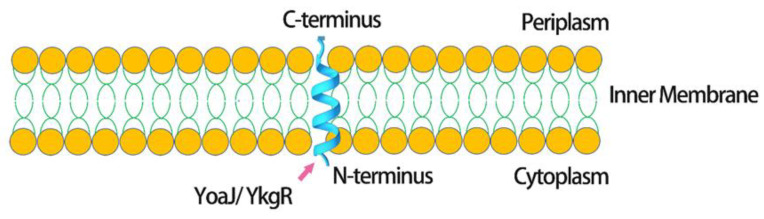
Location of the fusion tag (YoaJ/YkgR, small peptide, or protein) and recombinant peptide in *E. coli*, where OM is the outer membrane and IM is the inner membrane.

According to Fontaine’s explanation, the mechanism involving YkgR, which is one of the tiny transmembrane proteins, might be consistent with the transport mechanism of the Sec or YidC pathway. It can also be directed between the inner and outer membranes [122]. As shown in Figure 3, throughout the inner membrane, YkgR continues to translocate with the N-terminal centered in the cytoplasm and C-terminal centered in the periplasm. The YkgR tag can also be expressed at a high level in the periplasm. Therefore, the hypothesis of combining the YkgR tag with the target protein has been proposed. 

In this study, YoaJ/YkgR was used as a fusion tag in the periplasmic space of *E. coli* to generate peptides rich in disulfide bonds (Table 6). The position of YoaJ in *E. coli*, with its N-terminal in the cytoplasm and C-terminal in the periplasm, enables the peptides of interest to be fixed in the periplasmic space. Compared to other procedures involving *E. coli* periplasm, the current selection of leading peptides seems to improve the secretion efficiency and stability for the production of disulfide bridge-rich peptides and has been used to produce small peptides with favorable yields (Table 7) [123].

## 6. Co-Expression of Chaperones

Co-expression of molecular chaperones may be advantageous for increasing the solubility and folding of proteins. While chaperone co-expression increases the development of many monomeric and multimeric proteins, the effectiveness of this strategy seems to be protein specific [111,128,129]. Various factors lead to the failure of overexpressed proteins to fold into their original form, including the presence of molecular chaperones, lack of disulfide links, and/or post-translational modifications. Reducing cytoplasm deactivates disulfide bond formation [130]. In *E. coli*, the following two mechanisms lead to the breakdown of disulfide bonds: Thioredoxin mechanism involving trxB and the glutaredoxin network containing glutathione reductase, glutathione, and three glutaredoxins [131,132]. The use of *E. coli* strains that are deficient involves techniques to generate a less reductive cytoplasmic environment that promotes the formation of disulfide bonds with trxB, which contributes to sulfhydryl reduction [111,133,134]. Finally, purifying target proteins from the cytoplasmic protein pool is a relatively challenging task, as this compartment comprises the vast majority of the total cell protein.

The periplasm offers simple and cost-effective target protein purification from a slightly reduced pool of bacterial proteins compared to the cytoplasm. In addition, the oxidizing atmosphere of the periplasm promotes proper protein folding, and the in vivo cleavage of the signal peptide during translocation to the periplasm is more likely to deliver the authentic N-terminal of the target protein [135]. Signal peptides of prokaryotic and eukaryotic origin have been widely used for this function, although the existence of a signal peptide does not necessarily guarantee effective protein translocation across the inner membrane as certain other structural features are involved in conveying the membrane. Several techniques have been documented for improving the translocation of proteins to the periplasm, including the overproduction of the peptidase signal [136,137], reduction of protein expression levels to avoid overloading of the translocation mechanism, and co-production of several proteins involved in the membrane transport processes.

Protein folding in the periplasm can be induced by the overexpression of two types of enzymes: Protein disulfide isomerases (PDIs), which are predominantly observed in the periplasm and catalyze disulfide bond oxidation, and peptidyl-prolyl-cis-trans isomerases (PPIs), which catalyze X–pro bond isomerization [138]. At low temperatures, co-overexpression of DsbA and the heat-shock element improves the yield of correctly folded T-cell-receptor (TCR) fragments. Similarly, the coexpression of eukaryotic PDIs improves the yield of correctly folded pectate lyase C and bovine pancreatic trypsin inhibitor [121]. 

**Figure 4:**
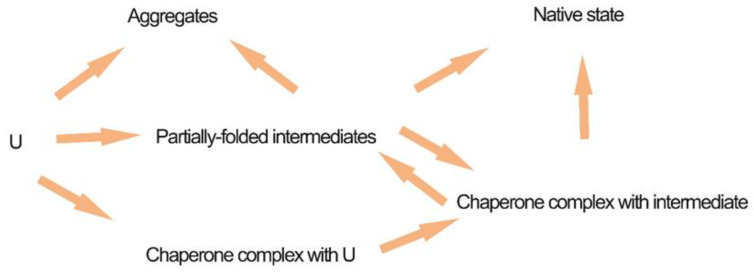
General outline of chaperone-mediated protein folding in a cell. U represents nascent polypeptide or newly synthesized protein. Chaperones include 40-, 60-, and 70-kDa heat shock proteins, protein disulfide isomerase, and peptidyl prolyl isomerase or trigger factor.

Co-overexpression of molecular chaperones is a potential method for avoiding the development of inclusion bodies. This technique is appealing; however, there is no certainty that chaperones can increase the solubility of recombinant proteins [22,139]. *E. coli* encode chaperones, some of which drive the process of protein folding, while others inhibit protein aggregation [134]. As soon as freshly synthesized proteins detach from the exit site of the *E. coli* ribosome, they are connected with chaperone with the cause factor [140,141]. Exposed hydrophobic patches on freshly synthesized proteins are shielded from unwanted inter- or intramolecular interactions by associating with the trigger factor, thereby preventing premature folding. After being released from the trigger factor, proteins may start or begin to fold into their native state [142]. Proteins in non-native conformations are vulnerable to aggregation and are used as substrates for DnaK and GroEL. By inducing aggregation and encouraging the proteolysis of misfolded proteins, DnaK (Hsp70 chaperone family) inhibits the development of inclusion bodies. The solubilization or disaggregation of proteins is regulated by a bi-chaperone mechanism involving DnaK and ClpB (Hsp100 chaperone family). GroEL (Hsp60 chaperone family) regulates the movement of proteins between soluble and insoluble protein fractions and is strongly involved in the development of inclusion bodies and disaggregation. Small heat shock proteins, such as lbpA and lbpB, protect heat-denatured proteins from irreversible aggregation and their expression is known to be correlated with that of inclusion bodies. The general outline is summarized in Figure 4. 

In many instances, simultaneous overexpression of chaperone-encoding genes and recombinant target proteins has proven to be successful. Co-overexpression of the stimulus factor in recombinants has inhibited the aggregation of mouse endostatin, human oxygen-regulated protein ORP150, human lysozyme, and guinea pig liver transglutaminase. Soluble expression is further enhanced along with the expression of the stimulus factor by the co-overexpression of the chaperone structures, namely, GroEL-GroES and DnaK-DnaJ-GrpE. The chaperone schemes are cooperative. The most desirable techniques include the co-expression of combinations of chaperones belonging to the chaperone families GroEL, DnaK, and ClpB, and the trigger factor aligned with the ribosome. 

## 7. Soluble and Insoluble Expression 

Numerous specific host strains have been developed to address the metabolic pressure associated with the high rates of protein production. The *E. coli* mutant strains added value to the soluble production of complicated recombinant proteins. C41(DE3) and C43(DE3) are mutants that enable the overexpression of certain globular and membrane proteins that cannot be expressed at high levels in the BL21(DE3) parent strain. The amount of cysteines present in *E. coli* cytoplasm is decreased by the trxB and glutaredoxin pathways. In Origami strains from Novagen, the disulfide bond-dependent folding of heterologous proteins is strengthened. Disruption of the *trxB* and *gor* genes encoding the two reductases allows disulfide bonds to be established in the *E. coli* cytoplasm. The *E. coli* strains negative for *trxB* (Novagen AD494) and *trxB/gor* (Novagen Origami) have been used for protein expression. The development of folding and disulfide bonds in the target protein is facilitated by thioredoxin fusion in thioredoxin-free (trxB) strains [28]. Overexpression of periplasmic foldase DsbC in the cytoplasm further promotes the formation of disulfide bonds. Batch culture is the easiest method for the development of recombinant proteins. In this method, all nutrients required for growth are supplied from the start, and only limited control can be exercised on the production during the cycle. Sometimes, this restriction contributes to the modifications in the growth medium, such as an increase in the pH and the concentration of dissolved oxygen as well as the degradation of substrates. Additionally, inhibitory compounds are derived from different metabolic pathways. The cell densities and output rates in batch cultivations are moderate. The concentration of energy sources that are modified is based on the rate of consumption in the fed-batch cultivations. Many other variables may also manage to achieve the optimum amount of output per biomass with respect to the target protein [5,135]. In fed-batch cultivations, the development of inclusion bodies may be observed by tracking shifts in intrinsic light dispersion via cytometric flow [143]. This enables the growth conditions to be controlled in real time as soon as inclusion bodies are observed even at low rates, and the formation of such bodies may be prevented theoretically [144]. Protein folding involves the presence of a particular cofactor. The addition of such cofactors or binding partners to the culture media may significantly increase the yield of the soluble protein. This phenomenon was demonstrated with a recombinant hemoglobin mutant, resulting in an increased aggregation of the soluble substance upon excessive addition of heme factor [145]. Similarly, a 50% improvement in the solubility of gloshedobin was observed when developed in the *E. coli* recombinant system with 0.1 mM Mg^2+^ [146]. The medium structure and optimization are essential considerations for the soluble expression of recombinant proteins. While this is achieved primarily by trial and error, it can be useful, nonetheless.

The overexpression of recombinant proteins in *E. coli* is commonly employed for manufacturing proteins in significant amounts owing to advantages, such as the development using cheap sources of energy, rapid deposition of biomass, suitability for fermentation with high cell density, and fairly quick output [14]. However, according to the data published by the Center for Eukaryotic Structure Genomics (CESG) (http:/targetdb.pdb.org/statistics/sites/CESG.html), only approximately 30% of the cloned targets in *E. coli*, among the total of 8048 targets, were presented in a soluble form, while the rest were presented either in a deteriorated form or as insoluble aggregates, classified as inclusion bodies [147]. While inclusion bodies cannot be used specifically for experiments involving protein activity, their insolubility offers a simple source of fairly pure protein, provided only certain proteins can be transformed to their natural and active conformation.

**Figure 5:**
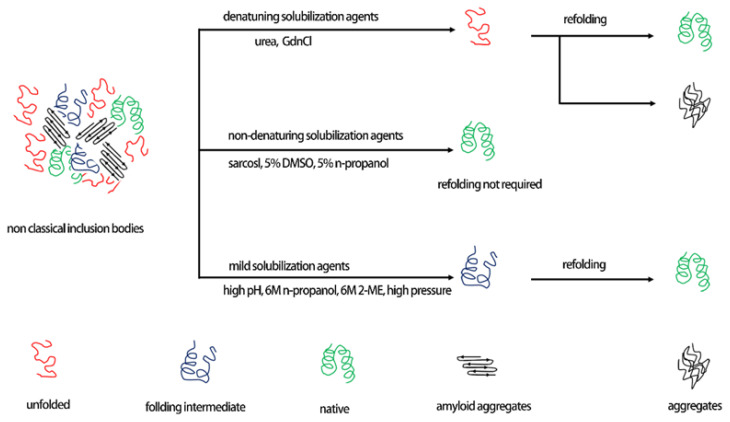
Model showing different solubilization methods used for the recovery of proteins from inclusion bodies.

To extract soluble active proteins from inclusion bodies, the insoluble inclusion bodies must be first solubilized in denaturants (Figure 5) and subsequently accompanied by a cycle of refolding (herein, referred to as the “one-step denaturing and refolding” procedure for comparison) [148]. This technique has been used for more than 20 years and is highly suitable for many bodily proteins, with approximately 40% being replenished into soluble and biologically active forms [138,149]. In this process, the inclusion bodies are denatured with a denaturing buffer containing either 6 M guanidine hydrochloride (GdnHCl), 8 M urea, or 0.3% sarkosyl (n-lauroyl sacosinate) [150]. However, in most situations, a substantial amount of precipitation occurs during the refolding of the proteins, resulting in a major reduction in the total yield of the desired proteins.

## 8. Purification 

**Figure 6:**
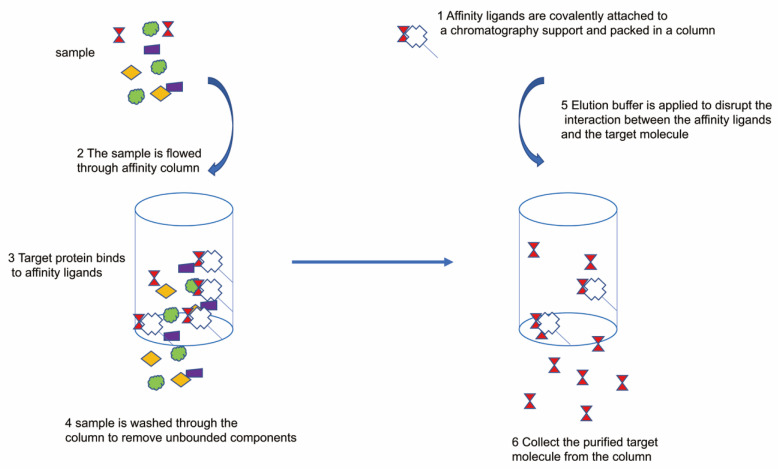
General protein purification process: protein of interest binds to column with resin, remove unbonded protein, and collect the target purified protein from the column by elution buffer.

As a chromatographic technique, IMAC offers the advantages of providing favorable and precise attachment, mild elution conditions, and the potential to monitor the selectivity by using low imidazole concentrations in chromatographic buffers [45]. The general purification process is simply illustrated in Figure 6. Those recombinant protein fused with tag can be purified by specific support resin in a column. For example, the construct of His6-insulin can be selectively purified by the Ni-NTA Agrose resin column. A broad variety of popular resins with different binding modes and abilities are available. However, all resins require difficult cleaning procedures. High-performance liquid chromatography can combine most purification steps; the most commonly utilized instruments are the ÄKTA systems from GE Healthcare [19]. The final quality of the protein can be controlled by monitoring the proportion of the recombinant protein with respect to the scale of the column; pollutants with lower affinity can interact with a greater abundance of the recombinant protein labeled with histidine. It is also useful to evaluate the sum of the soluble target protein to be loaded onto the board, which can be inferred from small-scale expression studies [151]. As a general rule, to optimize purity, the column is loaded with a small excess of the amount required to achieve the expected binding capability. While not mandatory, the implementation of such protein purification protocols with automated chromatography systems is reasonably straightforward, which has proven to be reliable, successful, and easy to use [21,152]. Protein and peptide affinity tags are widely used for the purification of recombinant proteins and native protein complexes. First, they offer 100- to 1000-fold purification of crude extracts without the need for undertaking any previous measures to eliminate nucleic acid or other cellular materials [153]. Secondly, the mild elution conditions allow the use of affinity tags valuable for purifying individual proteins, particularly complex proteins. Lastly, affinity tags permit the purification of diverse proteins using generic protocols as opposed to highly specialized procedures associated with traditional chromatography, compelling consideration for proteomics or structural genomics [1,154]. Most of the proteins and peptide affinity tags available have been produced during the last 20 years and can be divided into three groups based on the type of the affinity tag and its target: The first class includes epitope affinity tags used for peptide or protein fusion to tiny molecular ligands linked with a strong connection [155]. The hexa-histidine tag, for example, binds to an immobilized metal, while glutathione S-transferase protein fusions bind to glutathione that is bound to chromatographic resin [156]. The second class of affinity tags includes peptide tags that attach to an immobilized protein-binding partner on the chromatographic resin. For example, the calmodulin-binding peptide binds specifically to calmodulin, which allows the purification of proteins fused to the peptide that is attached to the calmodulin resin. The third type of epitope affinity tags may be classified in the second category, in which the protein-binding partner bound to the resin is an antibody that identifies a specific epitope of the peptide. Examples include the FLAG peptide for which an anti-FLAG antibody resin can be used. Such an abundant range of affinity tags for protein purification will render it challenging for a specific project to agree on the most suitable tag. While several, if not most, recombinant proteins are now processed and purified using affinity tags, just two to three tags have been compared in the few experiments testing affinity tags for protein purification. Thus, affinity tags are usually chosen depending on qualitative knowledge. To resolve this absence of a standardized analysis for affinity tags, we have previously measured the quality, yield, and expense by using two protein fusion and six short peptide affinity tags to purify two *E. coli*-expressed proteins [50]. We evaluated the capacity of the affinity resins to purify labeled proteins from extracts derived from yeast, *Drosophila*, and HeLa cells. Our tests, which demonstrate that the affinity tags are markedly different with respect to the efficacy of purification, offer a more robust foundation for choosing affinity tags. Several proteins and peptide affinity tags are now accessible that help distinguish proteins, which are expressed in a heterologous host, such as *E. coli*, or the purification by a labeled subunit of native complexes. In general, an ideal affinity tag exhibits the following properties and functions: (i) Enables the efficient purification of high-yield labeled proteins, (ii) can be used for any protein without disrupting its functions, (iii) can be positioned at any location in the protein (N-terminal, center, and C-terminal), (iv) can be used to purify protein produced in all host strains or expression methods, (v) can be used for the identification of recombinant proteins, and (vi) has resin attachments and elutes that are cheap, can be regenerated, and have strong flow properties. Notably, many affinity tags are available commercially that meet several, if not many, of those requirements. We analyzed two proteins and six peptide affinity tags, which met all of the above criteria, to purify recombinant proteins expressed in *E. coli* for their effectiveness [157]. We studied a subset of the capacity of such tags to purify proteins from three samples of eukaryotic cells. Our results report different purities of the derived proteins. We noted that epitope peptide tags, such as the FLAG peptide, yield the maximum-quality protein in *E. coli*, both for well-behaved and un-behaved polypeptides. Compounds derived from the bacteria, yeast, *Drosophila*, and HeLa include tags, such as the TAP tag involving the CBP peptide, which is widely used for the isolation of “normal” protein complexes, and the commonly used HIS tag that generates proteins with several contaminants. The Strep II (STR) tag was remarkably successful, delivering protein reliably and nearly as pure as epitope-based systems. By the purification of the affinity tag, we obtained fair yields of relatively high-purity DHFR. The truncated yeast polypeptide Gcn5 was extracted with a relatively weak purity and in small or virtually no quantity. Thus, while the purification by affinity tags allows for the use of general purification protocols that do not rely on the quality of the protein being purified, unfavorable properties of proteins, such as aggregation, can still affect affinity tag purification experiments [118,158]. Notably, the solubility of a recombinant protein is a minimum prerequisite for correctly folded nonmembrane proteins [159]. Soluble extracts that contain tiny aggregates of the labeled protein are not pelleted when centrifuged. Although these non-specific aggregates mask the affinity tag, as observed with the labeled Gcn5 polypeptides, resulting in the labeled protein being soluble and yet unable to attach to the affinity resin. Cost may be a critical parameter, in addition to purity and yield, when selecting affinity tags and resins, particularly for preparatory purification. We measured the cost of purifying 10 mg of a 30-kDa polypeptide using retail cost and resin efficiency details given by the distributors, to evaluate the price of different affinity resins. The columns MBP, Talon, Ni–NTA, and GST were the least costly, with resins costing $12–36 to purify the labeled polypeptide by 10 mg. To purify 10 mg of marked polypeptide, calmodulin (for CBP tag) and Strep-Tactin (for Strep II tag) were notably costlier at $114–293. The FLAG and HPC monoclonal resins are especially costly at $1000–5000 for the same volume of marked polypeptide due to their large unit resin cost and relatively small capacity (0.6 mg FLAG marked protein/mL resin versus 5–10 mg HIS tagged protein/mL resin) [115,160,161,162]. Choosing an attraction suffix specifically depends upon the criteria of the experiment. Experiments requiring large amounts of partly distilled content at a low cost might consider the HIS and GST tags, while experiments requiring small quantities with the maximum purity might consider the FLAG and HPC tags to overshadow their costs and minimal ability [107,163]. The Strep II tag appears to be an excellent candidate for affinity purification in general, as it is a short tag that produces high-purity proteins at a moderate cost with reasonable yields. One drawback attached to the use of the Strep II tag for protein crystallization in purifying proteins is that it interferes with the crystallization of a specific enzyme. This interference is not observed with the HIS or FLAG tags. Given that certain proteins have been successfully crystallized with the Strep II tag, it is not clear whether this tag adversely affects protein crystallization. These findings highlight the need for further comprehensive research examining the influence of protein crystallization by affinity tags. We consider that a mixture of HIS and Strep II tags enables rapid capturing of the tagged protein or protein complex from rough extracts by the Talon column, accompanied by polishing over the Strep-Tactin column. To promote the usage of the tags analyzed in this review, we prepared a suite of expression vectors that include the tags HIS, CBP, STR, FLAG, HPC, and CYD as single or double cleavable and non-cleavable tags for the production of individual polypeptides and polycistronic production of protein complexes in *E. coli*. We hope that our comprehensive study of affinity tags can help us make better choices for the purification of proteins. Comparing different tags utilizing a similar target protein has important benefits, and we recommend testing the potential affinity tags using DHFR in the future to enable clear comparisons with current tags. The knowledge presented in this study can also help to create new, smaller tandem affinity purification (TAP) tags as alternatives to the common combination of A–CBP proteins. X-ray crystallography of integral membrane proteins is difficult, as it needs significant expenditure of time and energy to locate a membrane protein that will produce diffraction-quality crystals. Nonetheless, we have shown that unproductive large-scale protein expression and purification can be reduced by the fluorescence-detection size-exclusion chromatography (FSEC), a fast pre-crystallization screening process in which mono-dispersion and goal protein stability are defined with only nanograms of unpurified protein. In this step, the target protein is fused covalently to GFP, and SEC analyzes the resulting unpurified fusion protein. While the GFP fusion technique was previously used to track the production of bacterial membrane proteins and screen detergents used for solubilization, our understanding of the use of the covalent GFP fusion and SEC techniques to examine mono-dispersiveness and fusion protein stability for protein crystallization is novel. In addition, pre-crystallization screening based on FSEC can be used with the previously mentioned GFP fusion method to develop cell lines with high levels of expression of promising constructs. In this review, the advantages and importance of covalent GFP fusion proteins and FSEC pre-crystallization screening with eukaryotic and prokaryotic membrane proteins have been illustrated. In such tests, small quantities of unpurified target membrane proteins were tested rapidly and easily for the extent of the position and expression, degree of mono-dispersity, average molecular mass, and detergent stability. In this analysis, size exclusion chromatography (SEC) was used instead of gel permeation, gel filtration, steric exclusion, exclusion, or gel chromatography. SEC is an entropically regulated separation strategy that relies on the relative size of a macromolecule or the hydrodynamic volume over the average pore size of the packaging. Importantly, SEC is a relative technique that involves column calibration to evaluate statistical average molecular weights and the distribution of polymers by molecular weight [101]. Absolute molecular weight measurements are therefore feasible with either an electronic light scattering device or a widely controlled on-line viscometer. The molecular conformation of these detectors and long-chain branching may also be evaluated by these methods. Along with the ability to examine molecular parameters, SEC is often useful for the preparatory fractionation of polymers and the isolation of small molecules from large polymeric or biogenic matrices during sample cleaning [164,165]. SEC is a standard and well-accepted methodology by which organic polymers and biopolymers are characterized. We have observed a higher usage of high-performance columns than traditional soft-gel packaging in life sciences. Owing to their speed and high resolution, high-performance columns are still preferred. Both silica- and organic-based packaging are used for aqueous SEC. Silica-based packaging tends to be favored for quality management and monitoring due to better performance (smaller particle size), shorter testing period, and more durable nature than organic-based packaging. Nevertheless, unintended contact during the labeling of solvents is a huge concern. Polystyrene gels are the choice of packaging for organo-soluble polymers. In this analysis phase, less attention is given to the theoretical aspects of SEC, including band extension, and the implementations are more heavily focused on the usage of electronic light-scattering detectors and viscometers. With these molecular weight-sensitive detectors, branching, molecular scale, and conformation can now be evaluated as a feature of molecular weight in one continuous experiment. Together with a concentration-sensitive detector, the usage of any such detector has significantly increased the sensitivity and precision of these tests.

## 9. Conclusions 

Proteins greater than 100 kDa are typically expressed in a eukaryotic system, while those less than 30 kDa are expressed in a prokaryotic system [166]. Mammalian cells, bacteria, or the baculovirus systems are selected for proteins involving glycosylation. The least expensive, easiest, and quickest protein expression can be performed in *E. coli*. The bacterium, however, cannot produce large proteins [167]. Additionally, for S–S-rich proteins and proteins involving post-translation modifications, *E. coli* is not the method of choice, as it cannot perform glycosylation and eliminate the S–S fragments. Eukaryotic proteins may also be toxic to the bacteria. Proteins with disulfide bonds are typically stored in the periplasm since the cytoplasm is very limited. The periplasm offers the advantages of simplicity and cost effectiveness for target protein purification from a slightly reduced pool of bacterial proteins as opposed to the cytoplasm. Furthermore, the oxidizing environment of the periplasm promotes adequate protein folding. Hence, the in vivo cleavage of the warning peptide in the periplasm during translocation is more likely to produce the authentic N-terminal of the protein. To this end, signal peptides of prokaryotic and eukaryotic origins have been successfully used. The two most utilized yeasts are *S. cerevisiae* and *P. pastoris*. For a low expense, yeasts can generate high protein yields as well as proteins greater than 50 kDa along with the extraction of signal sequences and glycosylation. Yeasts develop chaperonins to help fold those proteins and can process S–S-rich proteins [168]. The baculovirus system is a more advanced eukaryotic system than yeast, which can perform more complicated post-translational protein modifications. The most common system used to generate recombinant glycosylated mammalian proteins is the one developed using mammalian cells. Such cells can produce proteins greater than 50 kDa, accurately remove the signal chain, glycosylate, and even exhibit chaperonins. Such proteins observed in mammalian structures include factor VII, factor IX, factor IX, interleukin 2, human growth hormone, and TPA [169,170]. Transgenic animals can produce several valuable biopharmaceuticals, such as vaccines, antibodies, and other biotherapeutics [171,172]. Advances in molecular biology have been the primary guiding factor in the biopharmaceutical production of such substances and the development of high levels of protein. 

In conclusion, significant progress has been observed in many technical aspects of gene expression in *E. coli* and yeast. The advantages of prokaryotes and eukaryotes have ensured that they remain a valuable tool for the production of recombinant proteins for both basic research and commercial applications. 

## Figures and Tables

**Table 1:** Characteristics of *Escherichia coli* expression systems.

Advantages	Disadvantages
Rapid expression	Proteins with disulfide bonds are difficult to express
High yields	Production of unglycosylated proteins
Ease of culture and genome modifications	Proteins with endotoxins are produced
Inexpensive	Acetate formation results in cell toxicity
Rapid mass production and cost-effective	Proteins produced as inclusion bodies are inactive; thus, refolding is required.

**Table 2:** Advantages of yeast expression systems.

Advantages	Disadvantages
High yield	N or O-linked glycosylation pattern (different from higher eukaryote) Hypermannosylation Proteolytic degradation
Stable production strains	
Durability	
Cost-effective	
High-density growth	
High productivity	
Suitable for isotopically labeled protein production	
Rapid growth in chemically defined media	
Product processing similar to mammalian cells	
Can handle S–S-rich proteins	
Can assist in protein folding	
Can glycosylate proteins	

**Table 3:** Host strains and the produced recombinant proteins in them.

Host	Recombinant Protein	Reference
*E. coli*	venom proteins	[83,84]
*P. pastoris* *S. cerevisiae*	lipase r27RC rat protein disulfide isomerase	[85] [86]
*L. lactis*	Merozoite antigens	[87]

**Table 4:** Advantages and disadvantages of different locations of expression.

	Advantage	Disadvantage
Cytoplasmic	Higher expression level, simple plasmid construction	Inclusion body may be formed, unfavorable conditions for S-S bond formation, higher proteolysis
Periplasmic	Less proteolysis, improved folding, simple purification	Inclusion body may be formed, the signal does not always facilitate export
Extracellular	Least extensive proteolysis, simpler purification (fewer protein species), improved folding	Usually no secretion is observed, purification may be complex (protein dilution)

**Table 5:** Vectors based on different promoters used for heterologous protein production and their characteristics.

Vector	Induction (IPTG)	Level of Expression	Key Feature
lac	0.2 mM	Low to moderate levels; 15–30% of total cell proteins	Weak, regulated, suitable for gene products at very low intracellular level; comparatively expensive induction
Trc and tac	0.2 mM	Moderately high	High-level, but lower than T7 system; regulated expression still possible; comparatively expensive induction; high basal level
T7 RNA polymerase	0.2 mM	Very high; 40–50% of total cell proteins	Utilizes T7 RNA polymerase; high-level inducible overexpression; T7lac system for tight control of induction needed for more toxic clones; relatively expensive induction; basal level depends on used strain (pLys)
Phage promoter P_L_	Shifting the temperature from 30 to 42 °C (45 °C)	Moderately high	Temperature-sensitive host required; lower likelihood of “leaky” non-induced expression; basal level, high basal level by temperatures below 30 °C

**Table 6:** Peptides used in the current study.

Peptide	Disulfide Number
Beta-defensin	3
hepcidin	3/4
DkTx	6
ASPA	3

**Table 7:** Comparison of yields of proteins tagged with different signal peptides produced in the periplasm of *E. coli*.

Fusion Protein	Yield (mg/L)	Disulfide Bond Number	Tag Size (kDa)	Ref
YkgR-BD	~25	3	4	[123]
YkgR-Hep	~17	4	4	[123]
YkgR-DkTx	~8	6	4	[123]
YoaJ-ASPA	~5	3	2.69	[123]
YoaJ-BD	~10	3	2.69	[123]
DsbA-scFv	0.3		23.5	[124]
MBP-nanobodies	2.2		42	[125]
DsbC-Huwentoxin	0.7		23.5	[126]
pelB-human growth hormone	1.4		2.31	[127]
